# Azithromycin prevents implantation failure via up-regulation of leukemia inhibitory factor in endotoxemic pregnant rats

**DOI:** 10.22038/ijbms.2025.85311.18435

**Published:** 2025

**Authors:** Gonca Sonmez, Oznur Tufan Akarslan, Muhammed Hudai Culha, Tugba Melike Parlak, Burak Dik, Ayse Er

**Affiliations:** 1 Department of Genetics, Faculty of Veterinary Medicine, Selcuk University, Konya, Turkey; 2 Department of Pharmacology and Toxicology, Faculty of Veterinary Medicine, Selcuk University, Konya, Turkey

**Keywords:** Azithromycin, Cytokine, Implantation, Embryo implantation, Lipopolysaccharide

## Abstract

**Objective(s):**

Embryonic implantation is a complex and poorly understood process in which numerous cellular, hormonal, and molecular factors play critical roles. Infections in this process can result in pregnancy failure, such as implantation failure, infertility, and spontaneous abortion. Antibiotic use is necessary for infections. However, antibiotic use in pregnancy and the effect of the drug used on implantation are also conditions that must be considered. The implantation site is highly sensitive to lipopolysaccharide (LPS) and tumor necrosis factor (TNF)α, both of which can induce embryonic resorption. This study aimed to determine the effect of azithromycin (AZIT) on implantation failure, an important factor in early embryonic loss caused by LPS, by evaluating TNFα, interleukin (IL)-10, IL-2, and leukemia inhibitory factor (LIF) mRNA expressions in uterine tissue.

**Materials and Methods:**

The study involved twenty-six female rats, divided into four groups: Control, Sham, LPS, and LPS+AZIT. Lipopolysaccharide was administered intravenously on the 5th day of pregnancy in the LPS and LPS+AZIT groups. AZIT was administered intraperitoneally in the LPS+AZIT group simultaneously with LPS. TNFα, IL-10, IL-2, and LIF mRNA expressions were evaluated in uterine tissue three hours post-LPS administration.

**Results:**

Lipopolysaccharide administration increased the expression of TNFα and IL-2 and decreased the expression of LIF. AZIT prevented the LPS-induced increase in TNFα and IL-2 mRNA expression and the decrease in LIF mRNA expression, all of which are involved in implantation failure.

**Conclusion:**

AZIT may support the continuation of pregnancy by preventing the cytokine imbalance caused by infection at implantation.

## Introduction

Reproduction is essential for the survival of all species. Therefore, understanding the mechanisms that sustain pregnancy and fetal development is crucial. Implantation is a critical step in establishing and maintaining a healthy pregnancy in placental species such as mammals (1, 2). The blastocyst makes direct physiological and physical contact with the uterine endometrium during implantation. This period, known as the implantation window, typically occurs over a limited timeframe (3, 4). In rats, implantation begins on the 5^th^ day of pregnancy and is completed by the 7^th^ day (5). Defective implantation can lead to adverse outcomes such as infertility, spontaneous abortion, intrauterine fetal growth restriction, and preeclampsia. Reports indicate that implantation failure accounts for two-thirds of pregnancy losses (6). Furthermore, recurrent implantation failure (RIF), a multifactorial condition, affects 10-15% of couples undergoing in vitro fertilization (7). Tight regulation of the local immune environment is crucial for implantation (8). Consequently, functional dysregulation of the endometrial immune system is recognized as one of the primary pathophysiological mechanisms leading to RIF (7).

A critical system to prevent rejection exists during implantation, with the appropriate cytokine balance being a key component (9). Even with strong developmental dynamics and high-quality embryos, abnormal interleukin (IL) production can negatively impact implantation and lead to RIF (7). Cytokines are categorized as pro-inflammatory (Th1) or anti-inflammatory (Th2) based on their functions. Major Th1 cytokines include tumor necrosis factor (TNF), IL-2, interferon (IFN)-γ, and IL-1β, while Th2 cytokines include IL-4, IL-5, IL-10, and IL-13 (10,11). Disrupting the balance between excessively cytotoxic immune cell subtypes and decreased regulatory cellular elements increases inflammatory cytokines, disturbing the endometrial immune environment (7). Although inflammation is necessary for early implantation, excessive inflammatory factors in the implantation area during this period can cause embryo loss (12). Leukemia inhibitory factor (LIF), a member of the IL-6 cytokine family, is one of the most important cytokines in the female reproductive system (13). Leukemia inhibitory factor is involved in various processes, including blastocyst growth and development, uterine inflammatory responses to implanted embryos, trophoblast invasion, and embryo-endometrial interaction (14). Females with a non-functional LIF gene remain fertile, but their viable blastocysts do not implant or develop properly. However, when these blastocysts are transferred to wild-type pseudopregnant recipients, they can implant and grow to term (15). Intraperitoneally administered LIF antagonists have also been reported to cause implantation failure (14). 

Defects in maternal infection and inflammation-related cytokine expressions can lead to implantation failure. Therefore, LPS obtained from Gram-negative bacteria is used in low doses to create an inflammatory response in experimental pregnancy studies without affecting maternal survival. *Escherichia coli* (*E. coli*) has been reported to increase pro-inflammatory cytokines TNFα and IL-1β in pregnant animals’ circulatory system and intrauterine environment. IL-10, an anti-inflammatory cytokine, plays a crucial role against inflammatory stimuli by reducing pro-inflammatory cytokine expression in the implantation area, thus protecting against inflammation-induced pathology (16-18). Additionally, LPS can cause liver and kidney damage at high doses (19). Serum alkaline phosphatase (ALP), alanine aminotransferase (ALT), and aspartate aminotransferase (AST) levels are indicators of liver damage, while blood urea nitrogen (BUN) and creatinine levels indicate kidney damage (20).

Macrolide antibiotics are used during pregnancy for bacterial infections such as chlamydia, toxoplasmosis, and respiratory tract infections. They are also preferred as alternatives to beta-lactam antibiotics for treating infections in individuals allergic to them (21). Exposure to macrolide antibiotics during the first and third trimesters has not been associated with major malformations in the first trimester, nor has any association been shown with perinatal mortality, low birth weight, preterm birth, pyloric stenosis, or intussusception in the third trimester (22). Besides their direct antimicrobial effects, macrolide antibiotics (azithromycin, tylosin, tulathromycin) exhibit anti-inflammatory activity by reducing TNFα and increasing IL-10 levels (17, 23). Increased TNFα levels have been associated with decreased pregnancy rates, while IL-10 supports pregnancy continuation. Thus, maintaining a balance between TNFα and IL-10 is crucial for a successful pregnancy (17, 24).

Given the importance of implantation failure in early embryonic loss, this study hypothesizes that macrolide antibiotics with anti-inflammatory effects could prevent early embryonic loss through their impact on cytokines in uterine tissue. This project aims to determine the effect of azithromycin, a macrolide antibiotic, on implantation failure associated with LPS administration on the 5^th^ day of pregnancy by evaluating uterine TNFα, IL-2, IL-10, and LIF mRNA expressions.

## Materials and Methods

### Animals

This study utilized 26 female Wistar rats (196-278 gr, 11 weeks old), and the procedure was approved by the Selcuk University Experimental Medicine Application and Research Center Animal Experiments Local Ethics Committee (24.06.2022; 2022/21).

### Groups and applications

The animals were divided into four groups: Control (n=6), Sham (n=6), LPS (n=7), and LPS+AZIT (n=7). Female rats in estrus, determined by vaginal cytology, were housed with male rats overnight. The presence of sperm in the vaginal smear indicated mating and was considered day 0 of pregnancy. Lipopolysaccharide was administered intravenously via the tail at a dose of 160 µg/kg (17) to rats in the LPS (n=7) and LPS+AZIT (n=7) groups on the 5^th^ day of pregnancy. Azithromycin was administered intraperitoneally at 150 mg/kg (25) to the LPS+AZIT group simultaneously with LPS. The control group received no treatment, while the sham group received physiological saline intravenously (1 ml/kg) via the tail vein and intraperitoneally (32 ml/kg).

### Collection of samples

Rats were weighed on the first and last day of the experimental application. Blood (2 ml) was collected from the hearts of all animals under anesthesia (ketamine/xylazine, 60/10 mg/kg) three hours after LPS administration to evaluate albumin, ALP, ALT, AST, BUN, and creatinine parameters. The abdominal cavity was then opened, and one horn of the uterus was removed and stored at -80 °C for RNA isolation, cDNA synthesis, and qPCR analysis of TNFα, IL-10, IL-2, and LIF mRNA expression. Animals were euthanized by decapitation. To evaluate biochemical parameters, serum stored at -80 °C was used to measure albumin, ALP, ALT, AST, BUN, and creatinine levels using an autoanalyzer (DIRUI CS 4000).

### RNA isolation and reverse transcription reaction

Tissue samples were stored at -80 °C until RNA isolation. RNA isolation was performed using the commercial TRI Reagent (Sigma) protocol. Approximately 1 ml of TRI Reagent was used to homogenize the uterus samples (~50 mg). The homogenate was mixed with 300 μl of chloroform, and after centrifugation, 500 μl of isopropanol was added to the supernatant. The RNA pellet was washed three times with cold ethanol (70%, 70%, and 99%, respectively), and after sufficient drying, the RNA pellet was dissolved in 40 μl of Diethyl pyrocarbonate (DEPC) water. Nanodrop was used to determine the quality and quantity of total RNA. DEPC water was used for the blank. RNA at a 1 μg/10 μl concentration was run on 1% agarose gel electrophoresis. Ethidium Bromide (EtBr) stained RNA bands were evaluated on a UV transluminator by visual evaluation of band quality and the image taken from the imaging system. The concentrations of RNAs with A260/A280 and A260/A230 ratios between 1.8-2.0 and 2.0-2.2 were equalized using DEPC water to be 1 μg/2.5 μl. Then, DNAs were removed by treatment with DNase I (Thermo) enzyme. Reverse transcription was performed in 10 μl of a total reaction volume using iScriptTM cDNA Synthesis Kit (BioRad, USA). The nucleic acids (cDNA) were stored at −20 °C. 

### Real-time PCR analysis

The mRNA sequence information of the genes used in the study was obtained from GenBank (http://www.ncbi.nlm.nih.gov). Primer sequences were selected from previous studies (Table 1). Attention was paid to the fact that the primers did not form self and/or hetero dimer. For this purpose, the criteria specified by Wang and Seed (2003)(26) were taken into consideration. The accuracy of the primers and the region to be amplified were checked with the BLAST program in GenBank. The primers to be used were ordered from the B-Oligo company. The lyophilized primers were dissolved in NFW to a concentration of 100 pMol/μl. A small amount of cDNA from all samples was collected into an Eppendorf tube, and a pool was made. The efficiencies of the primers were calculated using cDNA pool dilutions.

The gene regions to be investigated were amplified in RT-qPCR using specific primers from the obtained cDNAs. The reaction was completed with 10 μl of NFW, containing 5 μl of SyberGreen master mix (BIO-RAD), 100 pmol of forward and reverse primers, and 2 μl of cDNA. The temperature profile of the reaction was set as 3 minutes at 98 °C, 35 cycles (15 sec at 95 °C, primer binding temperature 30 sec, 30 sec at 72 °C). Primer annealing temperatures of the genes were 60 °C for TNFα, IL-10, IL-2, LIF, and 57.6 °C for ACTB (actin beta, housekeeping). Melting curve analysis was performed. The data obtained from optical analysis in RT-qPCR with LightCycler Nano Software version 1.0 by Roche Diagnostics was recorded as Cq. The exact amount of NFW was used instead of cDNA as a negative control. The products obtained in RT-qPCR were run on 2% agarose gel electrophoresis, and the band sizes were observed to confirm that they were the correct products.

### Statistical analysis

The expression profiles of TNFα, IL-10, IL-2, LIF, and ACTB genes in uterine tissue were studied in triplicate. The Ct values​obtained by RT-qPCR results performed with primers with primer efficiencies between 90% and 110% were normalized with the Ct value obtained for ACTN (housekeeping) using the 2^-ΔCt^ method according to the criteria of Livak and Schmittgen 2001 (27). After normalization, differences between groups in cytokine expression values and biochemical data were evaluated by ANOVA and Tukey test (SPSS Version 22.0). *P*<0.05 was considered statistically significant in the results. Graphs were plotted using fold increases±SE values​ obtained with 2^-ΔΔCt^.

## Results

In this study, animals gained 14-38 grams during pregnancy, lasting from day 0 to day 5. While LPS administration did not affect albumin, ALP, BUN, and creatinine levels, it did increase AST and ALT levels (*P*<0.05, Table 2). Azithromycin administration alongside LPS prevented this increase, although the difference was not statistically significant (*P*>0.05).

**Figure 1:**
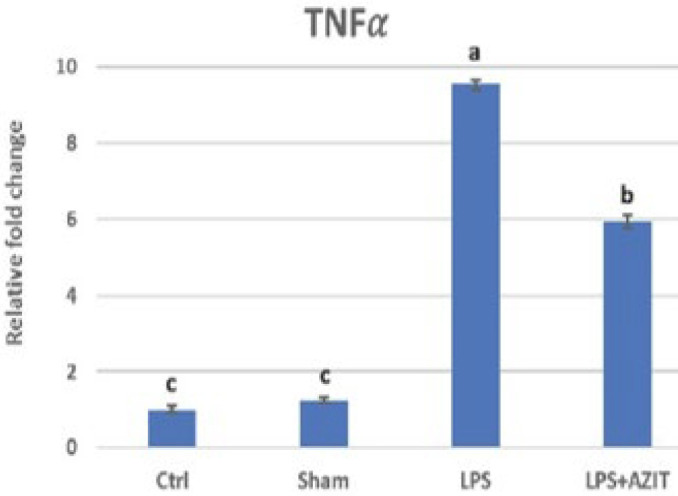
RT-qPCR analysis of TNFα mRNA expression in the rat uterus during the early embryonic period

**Figure 2:**
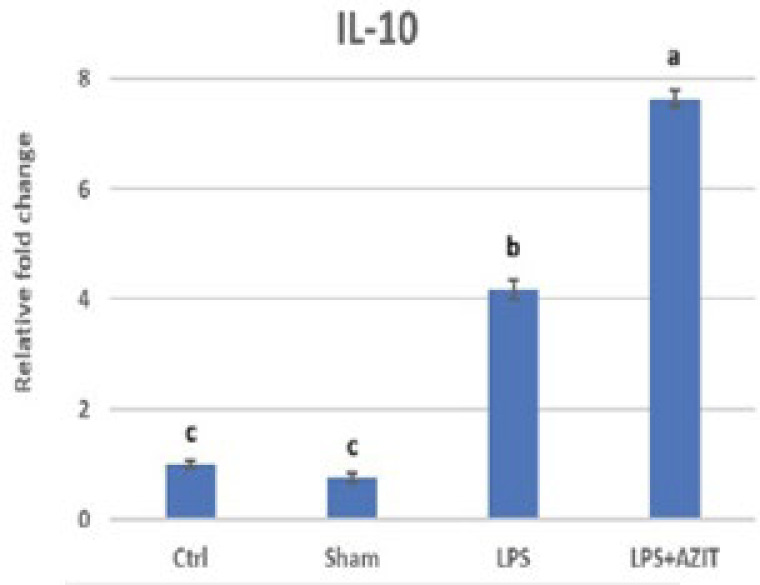
RT-qPCR analysis of IL-10 mRNA expression in the rat uterus during the early embryonic period

**Figure 3:**
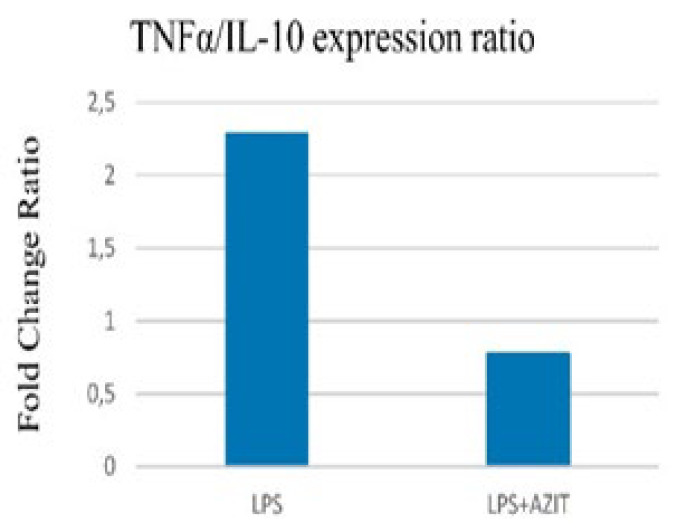
TNFα/IL-10 expression ratio

**Figure 4:**
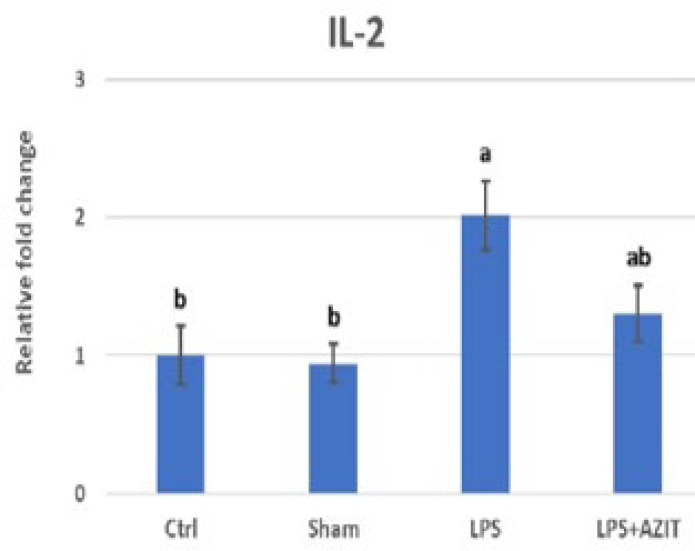
RT-qPCR analysis of IL-2 mRNA expression in the rat uterus during the early embryonic period

**Figure 5:**
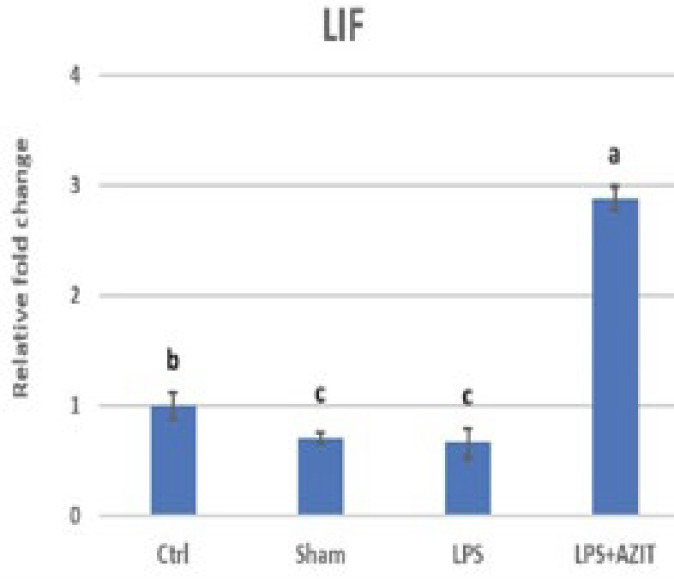
RT-qPCR analysis of IL-10 mRNA expression in the rat uterus during the early embryonic period

Compared to the control group, an increase in TNFα expression (9.53-fold), which plays an essential role in implantation failure, was determined in the LPS group, while there was no change in the sham group. In the LPS+AZIT group, the expression of this cytokine (5.93-fold) was partially inhibited compared to the LPS group (*P*<0.05, Figure 1). For the expression of IL-10, there was no difference in the sham group compared to the control group, while an increase was detected in the LPS (4.17-fold) and LPS+AZIT (7.65-fold) groups, with a greater fold increase in the LPS+AZIT group (*P*<0.05, Figure 2). The TNFα/IL-10 expression ratio was calculated as 2.29 in the LPS group and 0.78 in the LPS+AZIT group. This rate was determined to be quite low compared to the LPS group due to the application of AZIT together with LPS partially blocking TNFα expression and promoting IL-10 expression (Figure 3). IL-2 expression, another important cytokine in implantation failure, did not change in the sham group compared to the control group, and LPS caused an increase (*P*<0.05, Figure 4). In the LPS+AZIT group, the expression increase caused by LPS was partially prevented. A decrease in LIF expression was observed in the LPS group. In the LPS+AZIT group, the decrease caused by LPS was prevented, and the expression of LIF was promoted, which is essential in implantation (*P*<0.05, Figure 5).

## Discussion

Embryo implantation, a critical stage for placentation and pregnancy establishment, requires the mother’s immune system to adapt to prevent rejection of the semiallogenic fetus. This adaptation involves a range of responses from inflammatory to uterine tolerance (28-30). This complex molecular process involves the modulation of many factors, such as cytokines, growth factors, hormones, prostaglandins, matrix-degrading enzymes and inhibitors, and adhesion molecules (6). Cytokines play various roles in embryo implantation, placenta development, cytotrophoblast proliferation, vascular remodeling, trophoblast invasion, cell death, and inducing embryo tolerance (11).

Lipopolysaccharide administration on the 5^th^ day of pregnancy increased the expression of pro-inflammatory cytokines TNFα and IL-2, which can cause implantation failure if excessive (*P*<0.05, Figures 1 and 4). Azithromycin reduced these increases in TNFα (*P*<0.05) and IL-2 (*P*>0.05) levels. Exposure of pregnant rodents to LPS causes placental inflammation that contributes to embryonic reabsorption, fetal growth restriction, preeclampsia, fetal brain damage, and abortions due to changes in cytokine production (31). While an increase in TNFα and IL-1β expressions was determined in uterine tissues taken on the 4.5^th^ day after LPS application on the 3^rd^ day of pregnancy in mice, it was reported that IFN-tau applications starting six hours after LPS application had a protective effect on LPS-induced implantation failure by preventing these increases (32). In the study conducted by Gui* et al.* (33), it was stated that after mifepristone administration to rats, endometrial expression of IL-2, which is a harmful factor for implantation, increased on the 5^th^ day of pregnancy, and this played a role in implantation failure. It has also been stated that acupuncture application prevents implantation failure by reducing the expression of this cytokine. In this study, it is predicted that the application of azithromycin with LPS reduces the expression of TNFα and IL-2, which play an important role in implantation failure, and azithromycin may have a protective effect against events such as fetal death or abortion by preventing implantation failure.

In the present study, LPS surprisingly caused a fold increase in IL-10 expression. The higher fold increase in IL-10 expression in the LPS+AZIT group was found to be essential for maintaining pregnancy (*P*<0.05, Figure 2). While a decrease in IL-10 expression was determined in uterine tissues taken 4.5 days after LPS application on the 3^rd^ day of pregnancy in mice, it was stated that IFN-tau applications starting six hours after LPS application increased IL-10 expression (32). IL-10 applications have been shown to prevent LPS-induced abortions and reduce fetal death (16, 34). Azithromycin’s greater increase in IL-10 expression in this study suggests its potential to support healthy pregnancy continuation.

**Figure 6:**
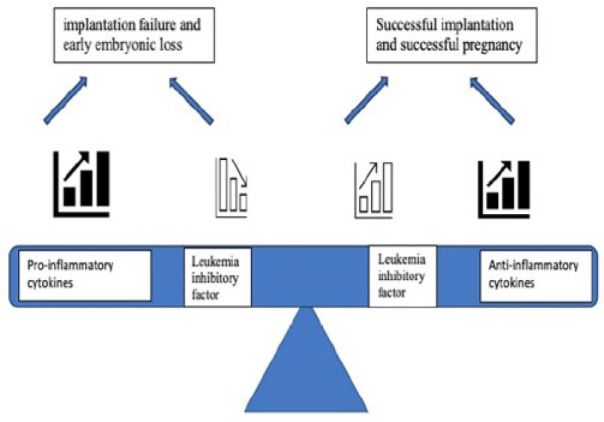
Balance of leukemia inhibitory factor and cytokines in implantation

Lipopolysaccharide significantly reduced LIF expression, but this decrease was prevented by azithromycin, and azithromycin promoted LIF expression (*P*<0.05, Figure 5). Although the reduction in the sham group is surprising, the lack of change in other cytokines important in the implantation site supports the view that this condition is temporary and will not result in failure. It was determined that LIF mRNA expression decreased significantly on 4.5, 5.5, and 8.5 days of pregnancy in polycystic ovary syndrome rats (35). It has also been stated that acupuncture may be a new tool for infertile women with LIF deficiency, as acupuncture promotes LIF expression to improve implantation (36). Leukemia inhibitory factor is crucial for embryo implantation, mediating processes like blastocyst growth, uterine preparation, decidualization, inflammatory responses, embryo-endometrial interaction, and trophoblast invasion (14). Blocking LIF with an antagonist decreases IL-10 expression, highlighting LIF’s importance in preventing implantation failure (37). Azithromycin administration with LPS may prevent implantation failure by increasing both IL-10 and LIF expression (Figure 6).

Lipopolysaccharide administration did not alter albumin, ALP, BUN, and creatinine levels (*P*>0.05) but increased AST and ALT levels (*P*<0.05, Table 2). Lipopolysaccharide administered at different doses and times during pregnancy did not cause any change in ALT, ALP, AST, creatinine, or BUN levels, or cause an increase in ALT, creatinine, or AST levels (38-40). The liver is crucial in the body’s defense against bacterial macromolecules, making it more susceptible to oxidative damage than the heart and kidneys (41, 42). High doses of LPS have been shown to significantly increase AST, ALP, and ALT values (19). However, in this study, the increase in AST and ALT values remained within the reference range due to low-dose LPS administration.

**Table 1:** Primer sequences, gene symbols, accession numbers, and product sizes (bp) for gene expression using RT-qPCR

**Gene symbols**	**Primer sequences** ** (5’- 3’)**	**Product sizes** ** (bp)**	**Accession numbers**	**Ref.**
**ACTB-F**	5'GGAGATTACTGCCCTGGCTCCTA3'	150	NM_031144.3	(Zhou* et al.* 2021) (43)
**ACTB-R**	5'GACTCATCGTACTCCTGCTTGCTG3'			
**TNF** **𝛼** **-F**	5'ATGGGCTCCCTCTCATCAGT3'	106	NM_012675.3	(Gholampour* et al.* 2019) (44)
**TNF** **𝛼** **-R**	5'GCTTGGTGGTTTGCTACGACG3'			
**IL-10-F**	5'CAGACCCACATGCTCCGAGA3'	141	XM_006249712.4	(Liu* et al.* 2018) (45)
**IL-10-R**	5'CAAGGCTTGGCAACCCAAGTA3'			
**IL-2-F**	5'TCTGCAGCGTGTGTTGGATT3'	142	NM_053836.1	This study
**IL-2-R**	5'TGGCTCATCATCGAATTGGC3'			
**LIF-F**	5′ATCAAGAGTCAACTGGCTCAACTCA3′	115	XM_008770359.3	(Zhou* et al.* 2021) (43)
**LIF-R**	5'TGTTGGGCGCACATAGCTTAT3′			

ACTB: Actin beta, TNF: Tumor necrosis factor, IL: Interleukin, LIF: Leukemia inhibitory factor

a, b, c: Different group letters are statistically significant (*P*<0.05). TNF: Tumor necrosis factor, Ctrl: Control, LPS: Lipopolysaccharide, AZIT: Azithromycin

**Table 2:** Effect of LPS and azithromycin application on serum biochemical parameters on the 5^th^ day of pregnancy in rat (mean±SE)

	**Albumin ** **(** **g** **/** **l** **)**	**ALP (U/** **l** **)**	**AST (U** **/l** **)**	**ALT (** **U/l** **)**	**BUN (** **mg/dl** **)**	**Creatinine ** **(** **mg/dl** **)**
**Control**	38±0.7	130±7	118±23^c^	51±3^b^	24±2	0.58±0.01
**Sham**	39±1.0	124±12	136±16^bc^	57±4^b^	25±4	0.58±0.01
**LPS**	39±0.9	130±6	236±26^a^	147±30^a^	34±2	0.65±0.02
**LPS+AZIT**	37±1.0	114±8	202±12^ab^	98±5^ab^	32±2	0.63±0.02

a, b, c; Different letters in the same column are statistically significant (*P*<0.05). ALP: Alkaline phosphatase, ALT: Alanine aminotransferase, AST: Aspartate aminotransferase, BUN: Blood urea nitrogen

a, b, c: Different group letters are statistically significant (*P*<0.05). IL: Interleukin, Ctrl: Control, LPS: Lipopolysaccharide, AZIT: Azithromycin

TNF: Tumor necrosis factor, IL: Interleukin

a, b: Different letters in groups are statistically significant (*P*<0.05). IL: Interleukin, Ctrl: Control, LPS: Lipopolysaccharide, AZIT: Azithromycin

a, b, c: Different group letters are statistically significant (*P*<0.05). LIF: Leukemia inhibitory factor, Ctrl: Control, LPS: Lipopolysaccharide, AZIT: Azithromycin

## Conclusion

It is speculated that azithromycin may prevent implantation failure caused by LPS-induced excessive pro-inflammatory cytokine expression and ensure pregnancy continuation in infections. Azithromycin’s anti-inflammatory effects may restore cytokine balance in cases of repeated implantation failure. Further studies are needed to consider target species and dose differences. Safe (non-teratogenic) anti-inflammatory antibiotics like azithromycin may benefit Gram-negative infections during pregnancy.

## Declaration

We have not used any AI tools or technologies to prepare this manuscript.
